# Amnesia Complicating Acute Rheumatism

**Published:** 1845-01

**Authors:** 


*Amnesia complicating Acute Rheumatism.*—A gentleman, by exposure to cold, contracted a fixed rheumatic pain in the neck, for which he only sought assistance eight days after the commencement of the attack. He had already been subjected to rheumatism, but had always recovered without medical assistance till now. He was ordered dry cupping over the part affected, and fifteen drops of colchicum wine every two hours. He was also desired to keep himself warm; but having, instead of this, exposed himself anew to cold, I was required to visit him several times on the next succeeding days, on account of several new and rather alarming symptoms, which led me to infer a metastasis of the rheumatism to the membranes of the brain. The patient could only utter, with extreme slowness, and stammering all the while, a few unconnected words, and had no power to name the articles of every-day life that were set before him, such as a book, a knife, a pen, &c.; the patient, nevertheless, could write their names down, and appeared perfectly to understand all the questions that were put to him concerning them. When the name of any thing placed before him was given him, he could also repeat it readily enough. The motions of the tongue appeared to be unaffected. The application of eight cups, with scarification behind the ears, succeeded by a blister, completely removed this condition. The rheumatism reappeared in the neck, and required further treatment. —*Ib., from Dr. Linsem, in Medicinische Zeitung.*

